# Impact of Cationic Stoichiometry on Physical, Optical and Electrical Properties of SrTiO_3_ Thin Films Grown on (001)-Oriented Si Substrates

**DOI:** 10.3390/ma17081714

**Published:** 2024-04-09

**Authors:** Marina Baryshnikova, Andries Boelen, Luca Ceccon, Vincent Herreman, Sean R. C. McMitchell, Christian Haffner, Clement Merckling

**Affiliations:** 1 Imec, Kapeldreef 75, 3001 Leuven, Belgium; andries.boelen@imec.be (A.B.); luca.ceccon@imec.be (L.C.);; 2 Department of Materials Engineering, KU Leuven, Kasteelpark Arenberg 44, 3001 Leuven, Belgium

**Keywords:** SrTiO_3_ template, STO, MBE, annealing, perovskites

## Abstract

In this study, we investigate the changes in the crystalline structure of MBE-deposited SrTiO_3_ layers on Si with different deviations from Sr/Ti stoichiometry as deposited but also after annealing at high temperatures (>600 °C). We show that as-grown 15 nm thick non-stochiometric SrTiO_3_ layers present surprisingly lower FWHM values of the (002) omega diffraction peak compared to fully stoichiometric layers of similar thickness. This can misleadingly point to superior crystalline quality of such non-stochiometric layers. However, thermal post-deposition anneals of these layers at temperatures up to 850 °C in oxygen show strong detrimental effects on the crystalline structure, surface and interface with the Si (001) substrate. On the contrary, the post-deposition anneals applied to the stoichiometric samples strongly improve the physical, optical and electrical properties of the epitaxial SrTiO_3_ thin films.

## 1. Introduction

SrTiO_3_ (STO)-templated Si wafers are widely used for the epitaxial growth of a large variety of multifunctional perovskite oxides and are often seen as one of the key routes for the heterogeneous integration of advanced devices [1,2] in CMOS-compatible technologies. For example, it has been demonstrated that SrTiO_3_ templates can be successfully used for growth of high-quality perovskites such as BaTiO_3_, SrRuO_3_ and Pb(Zr,Ti)O_3_ (PZT) for application in fast light modulators, electrodes, ferroelectric memory and others [3,4,5,6,7].

While the epitaxy of STO on Si has been achieved using techniques such as pulsed laser deposition [8], radio frequency sputtering [9] and atomic layer deposition [10], the highest crystalline quality is achieved by the molecular beam epitaxy (MBE) [11,12] approach which allows atomic-scale control of heterointerfaces and limits silicon oxidation; both features are essential for the epitaxial growth of STO.

Nonetheless, the growth of perfectly stoichiometric STO layers with high crystalline quality on Si by MBE remains a challenge. A conventional procedure consists in the use of a one-half monolayer (ML) of SrO which is thermodynamically stable on a Si surface and serves as a template for commensurate growth of crystalline STO [13] by a 45° rotation of the perovskite lattice with respect to the Si cubic lattice. Once the Si surface is passivated by a one-half ML of SrO, the growth of STO can be realized either by the careful adjustment of process conditions allowing the direct growth of the single crystalline layer [11,14] or by the recrystallization of initially amorphous material [15]. For both cases, the process window for formation of a stoichiometric material remains rather narrow; indeed, the process lacks a self-regulating mechanism due to the sticking coefficient of both cations being close to unity, and therefore, any excess or deficiency of Ti or Sr adatoms on the surface will be immediately incorporated into the epitaxial layer.

**Figure 1:**
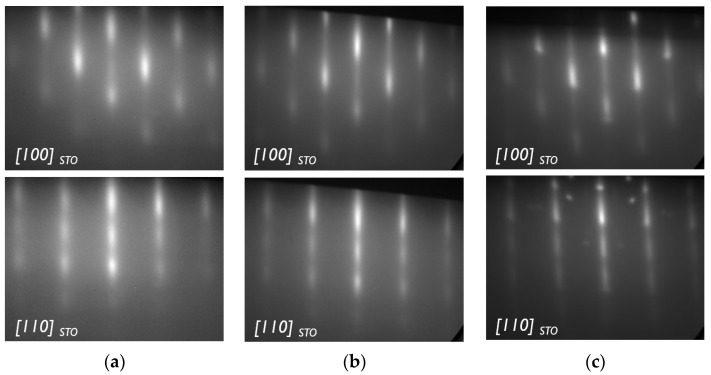
Typical RHEED patterns obtained from (**a**) Ti-rich, (**b**) stoichiometric and (**c**) Sr-rich STO layers.

The in situ and real-time reflection high-energy electron diffraction (RHEED) characterization technique is able to probe slight deviations in cation stoichiometry (up to 5%) by the appearance of specific surface reconstructions such as ×2 along [100]_STO_ or ×2 along [110]_STO_, for Sr-rich or Ti-rich SrTiO_3_ layers, respectively [16,17]. Larger deviations from cation stoichiometry could lead to streak extinctions along the [210] direction of STO in the case of a Ti-rich layer or additional diffraction spots in the case of Sr-rich layers, as shown in Figure 1c and [18], but are in general more difficult to interpret and evaluate.

For Ti-rich STO layers, as Ti is incorporated in the form of an amorphous material [19,20,21], it would lead to an increase in the background signal level (compare Figure 1a,b), but here again, it is difficult to accurately estimate the deviation from the ideal stoichiometry only based on background signal. If no additional analysis is performed, these layers can be mistakenly treated as stoichiometric STO layers on silicon.

In this study, we investigate the impact of high-temperature anneals on the crystal structure and surface morphology, as well as optical and electrical properties, of STO thin layers as a function of intentional deviations from cation stoichiometry. The interest is twofold: Firstly, STO templates on silicon are very often exposed to elevated temperature during growth of functional oxides, e.g., BaTiO_3_, and any uncontrolled changes in their structure might have an impact on the subsequent growing layer [22]. Secondly, it is reported that thermal annealing in an O_2_ atmosphere helps to reduce the oxygen vacancies typical for as-grown MBE layers [12,23] and in this way improves electrical properties like leakage current [24]; however, the impact of STO cationic stoichiometry was not studied in this case.

To investigate these points, we grew STO samples with different cationic stoichiometries which we annealed under various conditions. Afterward, changes in their crystalline structure and morphology were investigated using a variety of techniques, including TEM, ellipsometry and leakage current (I–V) measurements.

## 2. Materials and Methods

STO samples with different Sr/Ti ratios were grown using a MBE 49 tool (Riber, Bezons, France) on 200 mm p-type Si (001) substrates. The titanium was evaporated using an e-gun system with flux control based on a mass spectrometer combined with the use of feedback loop control on the power supply. Strontium flux was created using a double-filament Knudsen effusion cell, and molecular oxygen was supplied as the oxygen source during the growth. The growth temperature was kept at 350 °C with a growth rate of ≈1 nm/min. The MBE chamber was equipped with reflection high-energy electron diffraction (RHEED) to monitor the surface evolution during the growth in real time. All samples were epitaxially grown on Si substrates, and there were no indications of texture formation, as confirmed by phi-scans and selected area electron diffraction. More details on the growth procedure are reported in [25]. After the growth, the 200 mm wafer was diced into 2.5 × 2.5 cm coupons for subsequent annealing. The annealing was performed in a rapid thermal annealing system AS-One (Annealsys, Montpelier, France) in an oxygen flow at atmospheric pressure and 850 °C for 15 min, 30 min and 60 min.

The crystalline structure of as-grown and annealed STO layers was analyzed by high-resolution X-ray diffraction (HR-XRD), using an X’pert Pro diffractometer (PANalytical B.V., Almelo, The Netherlands). To extract in- and out-of-plane lattice parameters, 2θ-ω scans around STO (002) and (103) reflections were performed; to assess the mosaicity in the grown templates, the FWHM value of the (002) ω-peak was extracted. The variations in cation stoichiometry of STO (Sr/Ti ratio) were analyzed by means of Rutherford backscattering spectrometry (RBS) using a 1.5 MeV He^+^ ion beam and a silicon surface barrier detector. The surface morphology and roughness were investigated by atomic force microscopy (AFM) in pulsed force mode using Dimension 3100 tool (Bruker, Billerica, MA, USA).

For the microstructural investigation of the as-grown and annealed STO templates, a selected number of samples were studied with transmission electron microscopy (TEM). Firstly, TEM lamellas were prepared by focused ion beam (FIB). As a protection against ion beam damage during the FIB process, the samples were capped by a spin-on amorphous carbon (SOC) layer and thin ion-sputtered Pt layers. Then, an analysis of the samples was performed using a Titan^3^ G2 60-300 system from FEI (Thermo Fischer Scientific, Waltham, MA, USA) at a voltage of 200 kV. Different image conditions were applied such as high-resolution TEM, two-beam bright-field (BF) TEM, high-angle annular dark-field scanning transmission electron microscopy (HAADF-STEM), annular bright-field STEM (ABF-STEM) and dark-field STEM (DF-STEM).

The optical properties, such as refractive index and extinction coefficient, were studied by spectroscopic ellipsometry (SE) using a RC2 ellipsometer (J. A. Woollam, Lincoln, NE, USA). Data were obtained under angles between 45° and 85° with steps of 5° and an acquisition time of 3 s. Accessory CompleteEASE software (version 6.65) was used to fit and extract the optical indices, and the model used for this fitting is described in more detail in Appendix A.

Finally, to measure the out-of-plane leakage current in the STO, a 100 nm thick continuous Pt contact was sputtered on the backside of the samples, and circular Ti/Au contacts were thermally evaporated on the topside. Here, Ti(20 nm)/Au(30 nm) contacts with a diameter of 200 µm were obtained through an e-beam lift-off process using an MMA/PMMA double layer as a resist. Leakage I–V curves were obtained using a Keithley 4200-SCS parameter analyzer connected to a Summit 11000 probing station. The Pt back-contact was grounded, while a Ti/Au contact close to the center of the sample was biased first from 0 V to −5 V and back to 0 V, and then from 0 V to +5 V and back. A step of about 30 mV and a delay of 1 s were used.

## 3. Results and Discussion

### 3.1. Impact of Cation Stoichiometry

To estimate the difference in the annealing behavior of STO templates, we prepared three samples with different Sr/Ti ratios: 0.70, 0.96 and 1.11. The reason for choosing these particular stoichiometry values for this investigation was that for Sr/Ti < 0.7 and Sr/Ti > 1.1, a clear deterioration of RHEED patterns was observed. For convenience, we will refer to these layers as Ti-rich STO, stoichiometric STO and Sr-rich STO, respectively. The results of the RBS, XRD and AFM characterizations of the as-grown samples are summarized in Table 1.

Among all as-grown samples, it is the stoichiometric sample that presents the smoothest surface with an average roughness of R_a_ = 0.21 nm. For Ti-rich and Sr-rich STO, the roughness is R_a_ = 0.36 nm and R_a_ = 0.52 nm.

The extracted in- and out-of-plane lattice parameters show that the grown STO layers in all cases are compressively strained. Since the epitaxy of STO on Si includes its 45° lattice rotation [13], to judge the initial strain state, one needs to compare STO in-plane lattice parameters with half of the diagonal of the Si lattice, equal to 3.84 Å. For the Sr-rich sample, the in-plane lattice parameter almost matched the corresponding Si value, indicating possibility of the formation of a fully strained layer. For stoichiometric and Ti-rich layers, it was slightly larger, 3.87 Å. Therefore, those layers could be considered as partially strained in relation to the Si substrate. The calculated out-of-plane parameters are different for all STO samples ranging from 3.92 Å to 3.95–3.96 Å. The smallest value corresponds to stoichiometric layer, while the largest values belong to the samples with Sr or Ti deficiency. This observation suggests that deviations in cationic stoichiometry lead to the formation of STO with larger lattice parameters as a result of more pronounced lattice distortions, which is also reported in previous work [19,21,26].

One striking difference between the stoichiometric sample and the other two samples though is the FWHM value of the (002) ω-peak. For the Ti-rich STO, it was equal to 0.4°, which was almost three times lower than that for the stoichiometric sample. This fact, however, can hardly be attributed to better crystallinity and lower defect density because, as it was reported, a large excess of Ti tends to be incorporated into the STO layer in the form of an amorphous TiO_x_ phase [19]. However, it is possible that the presence of amorphous TiO_x_ in the Ti-rich sample affects the mechanism of strain relaxation of crystalline STO, leading to more uniform strain distribution across the layer thickness. An excessive amount of Ti could increase the critical thickness of non-stoichiometric STO, postponing the onset of plastic relaxation. For stoichiometric STO on Si, the critical thickness is in the range of 5–10 nm [12,27], which is below the thickness of our templates. Thus, the higher FWHM value of our stoichiometric sample can be due to the plastic relaxation and presence of strain gradients at the interface area. The Ti-rich sample in this case could still be more elastically strained, which would explain the larger out-of-plane lattice parameter and narrower (002) ω-peak.

For the Sr-rich STO, the FWHM is 0.6°, which is also much lower in comparison to stoichiometric samples. In this case, excess Sr can be incorporated as crystalline SrO planes forming stacking faults [18] or even Ruddlesden–Popper phases [21]. However, the effect on overall relaxation appears to be similar to that in the case of Ti excess. The out-of-plane lattice parameter is larger than the one from stoichiometric sample, which together with lower FWHM points to a different strain state of the layer.

### 3.2. Impact of Annealing on STO Microstructure

**Figure A3:**
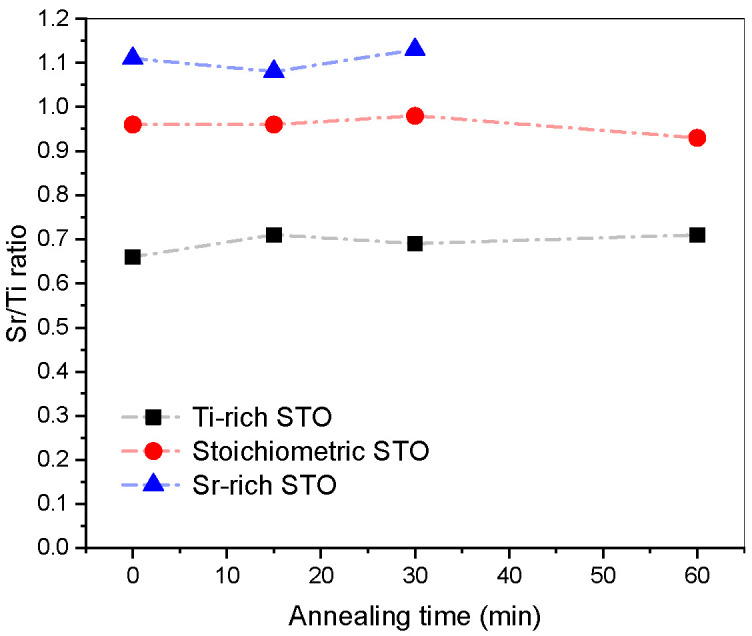
RBS results after different annealing times for STO samples with different Sr/Ti ratios.

After the initial characterization of as-grown samples, annealing experiments in an oxygen atmosphere were performed for different dwell times. For the annealing, we used 850 °C as lower temperatures led to a much reduced impact on the crystalline structure. At the same time, higher temperatures caused sample deterioration due to strong oxidation at the interfaces. It was confirmed from RBS analysis that cationic stoichiometry remained constant even after annealing for 60 min (Appendix B, Figure A3) for all samples. This observation contradicts some literature results [9,28] that indicate possibility of Sr depletion of samples heated to 850–900 °C.

**Figure 2:**
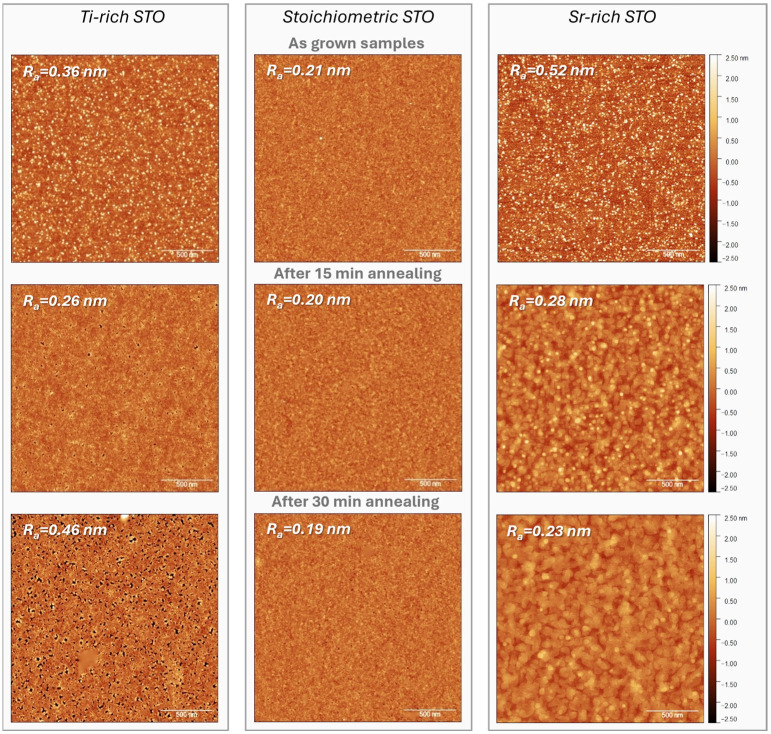
AFM images before and after annealing of samples with different Sr/Ti stoichiometries (2 × 2 µm range).

The results of the AFM investigation show that annealing had a different impact on surface morphology that depended on STO stoichiometry. For the layer with good stoichiometry, the roughness R_a_ remained around 0.2 nm even after annealing for 30 min. For Ti-rich STO, the roughness first slightly reduced to R_a_ = 0.26 nm after annealing for 15 min, but with longer annealing time, the roughness then increased up to R_a_ = 0.46 nm. Figure 2 also shows that the sample began to develop small pin holes after 15 min of treatment. These defects were also noticeable in the stoichiometric sample, but at much lower density and only after 30 min of annealing. Similar defects after thermal treatment have also been observed in [29].

The surface of the Sr-rich sample, on the contrary, showed a considerable improvement with annealing time. Already after 15 min of treatment at high temperature, the R_a_ value dropped to 0.28 nm, going further down to 0.23 nm upon annealing for 30 min. For these samples, in the corresponding AFM images in Figure 2, one can distinguish steps of 0.4–0.8 nm height that grew in height with the increase in annealing time. The height of the steps roughly matches the height of 1–2 monolayers of the SrTiO_3_ unit cell, which can indicate that annealing caused the formation of SrO- or TiO_2_-terminated surface structures.

**Figure 3:**
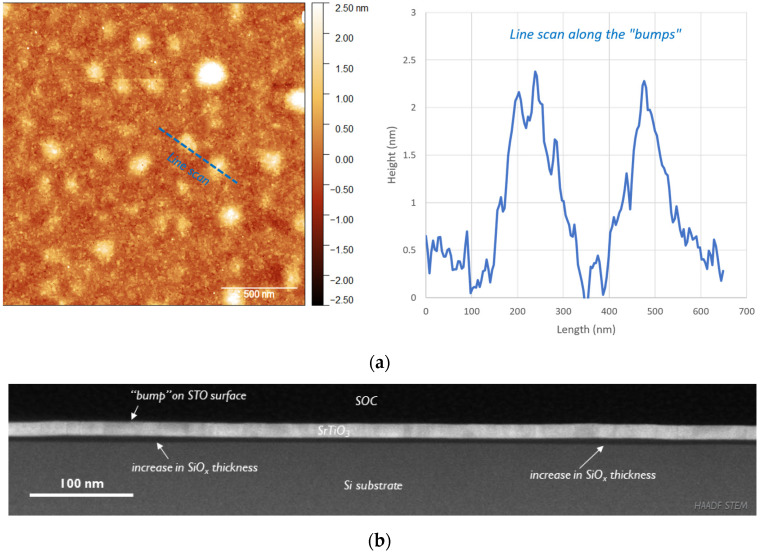
AFM image of (**a**) stoichiometric STO sample annealed for 60 min with line-scan across a “bump” and (**b**) cross-section HAADF STEM image of the same sample.

The AFM investigation also showed that long (>30 min) annealing time has a detrimental effect on STO templates. For the samples annealed for 60 min, the surface starts to develop multiple “bumps” of 2–8 nm height and 100–300 nm in diameter (Figure 3a). TEM investigation allowed us to find the origin of these bumps. In Figure 3b, in the cross-section image of the full layer stack at low magnification, one can notice a thickening of the interfacial SiO_x_ layer in some areas which causes the STO film on top to slightly elevate. Thickness and length measurements in these areas confirm that they match the dimensions of the bumps observed in AFM inspection. It is possible that the increase in SiO_x_ thickness in certain locations is linked to the poorer film quality in those regions. For example, the presence of threading dislocations, grain boundaries or other crystalline defects could facilitate oxygen diffusion towards the interface, leading to a locally higher oxidation rate and eventually thickness non-uniformity.

Along with the surface morphology, post-growth thermal anneals in oxygen also present a strong impact on the crystalline structure of STO templates, resulting in drastic changes in FWHM values and the intensities of (002) ω-peaks as well as lattice parameters. To better illustrate that these changes have a direct dependency on the template stoichiometry, we also considered results from an additional Ti-rich SrTiO_3_/Si(001) sample with the intermediate Sr/Ti ratio value of 0.86.

**Figure 4:**
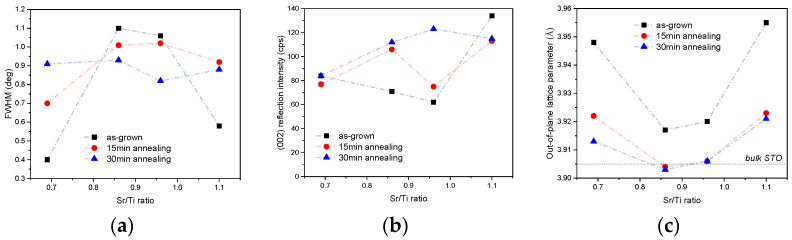
Results of (**a**) FWHM of (002) ω-peak measurement, (**b**) (002) reflection intensity and (**c**) out-of-plane lattice constant after annealing.

Looking at the outcomes of this XRD investigation presented in Figure 4, the STO samples can be split into two groups based on the changes in their structure upon annealing. The first group consists of the stoichiometric sample and the additional sample with a relatively small excess of Ti. These samples showed an overall improvement in their crystal quality with anneal time: as shown in Figure 4a,b, for these STO layers, annealing for 30 min led to decrease in the FWHM of the (002) ω-peak from ≈1.1° to ≈0.8° and an almost two times increase in the intensity of the (002) reflection. The second group of samples, consisting of STO with Sr/Ti of 0.70 and 1.11, showed the complete opposite behavior upon the application of the thermal treatment under oxygen. Their FWHM values rapidly increased from 0.4–0.6° to 0.9° while the diffraction peak intensity remained almost constant.

For both groups of samples, annealing at high temperature promoted strain relaxation in the layers, as can be seen from the ubiquitous reduction in the out-of-plane lattice parameter in Figure 4c. For the samples from the first group, the out-of-plane lattice parameter after 15 min annealing was already equal to the value corresponding to the theoretical lattice constant of STO-3.905 Å. For the non-stoichiometric samples from group 2, the decrease in lattice parameters also indicated a relaxation process; however, the values remained higher than 3.905 Å, indicating a presence of some residual strain or other structural differences like the occurrence of Ruddlesden–Popper phases in the SrTiO_3_ perovskite layer [21]. This highlights the two different strain mechanisms: stoichiometric samples seem to anneal out defects that cause the strain gradients, and non-stoichiometric seem have these outgrowths/interstitials that alter the intrinsic lattice constant and, therefore, cannot be annealed out.

It is important to note here that as-grown MBE STO samples often lack oxygen that is known to lead to the volume expansion of the unit cell common for perovskites [30]. Based on our results, it is difficult to estimate the contribution of this effect on the extracted values of the lattice constant as our layers are strained in relation to silicon. However, we can confirm that the initially deposited templates are oxygen-deficient by leakage current measurements discussed later in this paper.

The outcomes of AFM and XRD investigations demonstrate that annealing for up to 30 min contributes to smoothening of stoichiometric and slightly Sr-rich STO and overall increase in crystallinity of samples with a close to stoichiometric Sr/Ti ratio; however, it has a damaging effect for samples with Sr deficiency. However, further increasing the annealing time up to 60 min, independent of the sample stoichiometry, had a negative effect on the template’s microstructure due to interfacial reactions occurring at the STO/Si boundary.

**Figure 5:**
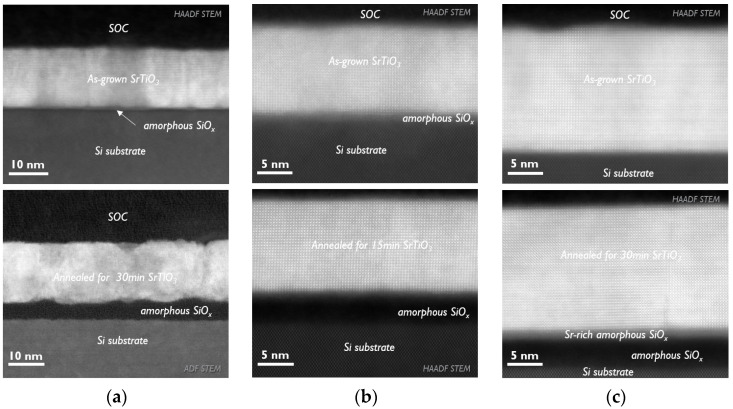
STEM images of STO templates with (**a**) Sr/Ti = 0.70, (**b**) Sr/Ti = 0.96 and (**c**) Sr/Ti = 1.11 before and after annealing.

These AFM and XRD investigations are supported by TEM analysis. As shown in Figure 5b, the as-grown stoichiometric sample exhibits overall good epitaxial quality despite the presence of thin interfacial SiO_x_. The layer looks uniform, although some blurriness in the STO layer could indicate a certain level of crystal distortion. Annealing just for 15 min helps to improve the crystallinity and surface roughness of the stoichiometric sample. In corresponding the TEM image, one can observe an almost flat top interface and atomic columns with very sharp contrast representing a high level of crystal order. It is interesting to notice that regardless of the increase in the interfacial SiO_x_ layer to 5 nm, the STO layer could preserve its thickness with improved crystalline quality.

**Figure A4:**
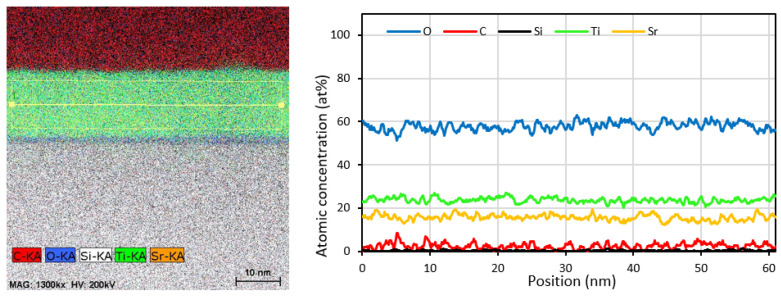
EDS scan along Ti-rich STO layer.

The Ti-rich sample before annealing (Figure 5a) looks much less uniform due to poorer crystalline quality. The contrast variation in HAADF images is proportional to the Z-number of the elements composing the layer. Therefore, darker areas visible in STO layer could indicate Ti-rich regions. The EDS analysis (Appendix B, Figure A4) along the layer, however, did not capture these local variations in Ti or Sr concentrations, which means that excess Ti is distributed uniformly along the STO, at least on the scale of a few tens of nanometers. The darker areas visible in the HAADF-STEM image are therefore due to a poorer crystallization level, the presence of pores or local variations in the TEM lamella thickness. The annealing of the Ti-rich sample is clearly detrimental to the STO layer. It creates very rough interfaces, and on the surface, many pits of up to 5 nm depth are visible, perfectly matching the AFM characterization (Figure 2). At the bottom heterointerface, numerous small protrusions of amorphous SiO_x_ into the STO layer are visible. It is then not a surprise that such a deterioration of the layer microstructure resulted in an almost two-fold increase in the FWHM of the (002) ω-peak, but it is still difficult to reliably judge the underlying mechanism leading to such dramatic changes in the layer’s microstructure. One possible hypothesis is that the excess of amorphous TiO_x_ being evenly distributed in the layer could promote layer recrystallization, binary oxide outgrowth and grain formation, and faceting at high temperatures. This kind of behavior is often observed in pure TiO_x_ films [31]. However, we could not detect any reflections corresponding to crystalline TiO_2_ in our layers during XRD analysis.

The cross-sectional TEM of the as-deposited Sr-rich sample (Figure 5c) looks better than the stoichiometric sample in terms of crystallinity. It also offers a much sharper interface between the Si and STO layers with only a very thin interfacial layer of SiO_x_ present in some areas. A more detailed analysis of the images indicated the presence of a few stacking faults in the SrTiO_3_ layer. The brighter contrast of the atoms composing these areas suggests that these are Sr-terminated defects. These observations are in alignment with the results published in [18,21,26] and confirm the incorporation of excess Sr in the form of vertical SrO planes. The incorporation of extra SrO planes leads to additional lattice expansion as measured in XRD and is well justified in [26]. Moreover, since the annealing did not change the Sr/Ti ratio of the sample, we can conclude that it did not affect the concentration of such stacking faults either, which can explain the larger lattice constants of Sr-rich STO after annealing. Another interesting observation is that annealing caused the formation of an additional Sr-rich layer at the bottom interface with the Si substrate which produces a clear difference in contrast in the HAADF image. Since the formation of such a layer is detected only for the Sr-rich sample, it indicates that a certain amount of Sr excess is incorporated as interstitial atoms and diffuses towards the bottom interface upon annealing. It is known that Sr, unlike Ti [32,33], has a high diffusion coefficient in SiO_2_ [34]. Therefore, it is logical to assume that if Sr atoms are weakly bonded to the STO lattice as in the case of their interstitial position, they can easily diffuse into SiO_2_ at high temperatures, forming an additional interfacial layer.

### 3.3. Impact of Annealing on STO Optical and Electrical Properties

It is known that oxygen stoichiometry is important for ensuring the best optical and electrical properties of STO layers [26,35,36]. However, very little information can be found on the impact of cationic non-stoichiometry. In this part, we discuss how basic electrical and optical properties such as leakage current, refractive index and light absorption were affected by deviations from the Sr/Ti ratio and annealing.

**Figure 6:**
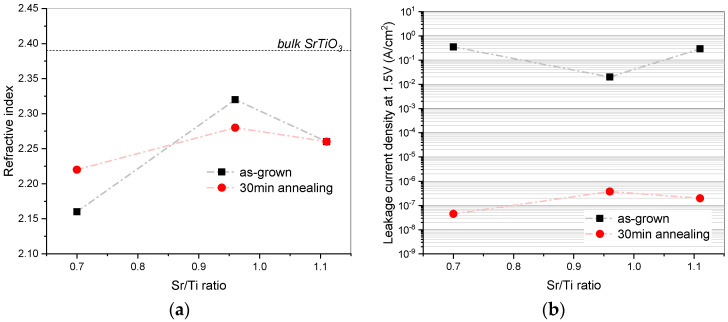
Dependance of (**a**) refractive index and (**b**) leakage current density at +1.5 V on STO stoichiometry before and after annealing.

The evolution of the refractive index, n, at 633 nm was calculated from ellipsometry measurements and is plotted in Figure 6a. Among all the samples, the stoichiometric STO layer had the highest n, both before and after annealing. The smaller refractive index of the Ti-rich layer cannot be attributed to the excess of Ti, as the n value of TiO_x_ lies in the range of 2.5–2.7 depending on its structure. Therefore, the lower refractive index of the Ti-rich sample is most likely due to poorer layer morphology in comparison to the other samples. For the Sr-rich STO, the refractive index is just slightly smaller than the value for stoichiometric STO. Since the layer microstructure in this case was comparable to that of the stoichiometric sample, the reduction in n could be due to an excess of SrO which has refractive index of ≈1.9. It is also interesting to note here that for all the samples considered in this study, the refractive index remained lower than that for bulk SrTiO_3_ (n_633nm_ = 2.39). Considering the small thickness of the samples, the lower refractive index was most likely caused by the proximity of the interface with SiO_x_ (refractive index ≈ 1.4), as has been reported in [37].

**Figure A5:**
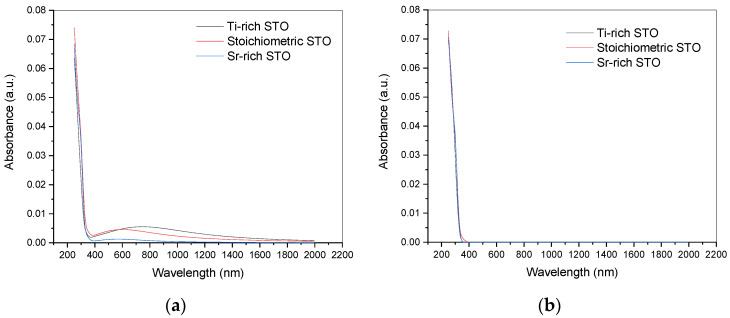
Optical absorption spectra of STO layers with different stoichiometries (**a**) before and (**b**) after annealing.

From the ellipsometry measurements, we could also extract the absorption spectra, which indicates that the as-grown films are not fully transparent in the 400–1500 nm range, but this was improved after annealing in oxygen. These observations are in good agreement with the previously reported experimental results where the increased absorption was attributed to the presence of oxygen vacancies [35,36] (Appendix B, Figure A5). Annealing helped to recover the oxygen concentration in the films and thereby increase their transparency.

The electrical properties were assessed by means of leakage current measurements and are reported as current density values at 1.5 V in Figure 6b. As expected, the as-deposited samples were quite conductive due to the oxygen deficiency inherited from the chosen deposition technique. An additional deviation from the stoichiometry in Sr- or Ti- rich samples caused even higher leakage currents. It is possible that the excess of Ti or Sr incorporated in the interstitial or substitutional positions might create additional leakage paths. Annealing in an oxygen atmosphere caused a great improvement in electrical properties. The measured leakage current at 1.5 V was at the detection limit of tool, indicating the highly insulating properties of the films.

## 4. Conclusions

The goal of this study was to investigate the impact of cationic deviation from stoichiometry in STO templates on their crystalline quality after high-temperature annealing. One of the important findings of this work is that if no special attention is paid, Ti-rich STO templates can be mistakenly treated as layers with very good crystalline quality as they show very low values of the FWHM of the (002) ω-peak. High-temperature annealing in an oxygen atmosphere significantly improves the quality of stoichiometric layers, leading to better crystallinity and surface roughness. For the Ti-rich STO, thermal annealing in oxygen had an adverse effect leading to the formation of films with a very non-uniform microstructure and a degraded surface. The annealing time also plays a key role and perfectly illustrates the importance of kinetic energy in such a material system. For 15 nm thick stoichiometric STO layers, high-temperature annealing for 15–30 min is enough to initiate lattice relaxation and benefit from an improvement in the crystallinity, but longer annealing leads to interface degradation. For Ti-rich STO, a negative impact was already observed after 15 min.

The importance of cationic stoichiometry was also demonstrated for such properties as film transparency, refractive index and leakage current. However, it has been shown that it is primarily significant for the as-grown layers. Annealing in oxygen significantly improved the optical and electrical characteristics of the films, independent of their Sr/Ti ratio.

## Figures and Tables

**Table 1:** Results of RBS, XRD and AFM analysis of as-grown STO samples with different stoichiometries.

	Ti-rich STO	Stoichiometric STO	Sr-rich STO
Thickness (nm)	17	13	18
Sr/Ti ratio from RBS	0.70	0.96	1.11
(002) ω-peak FWHM (deg)	0.4	1.1	0.6
Out-of-plane lattice parameter (Å)	3.95	3.92	3.96
In-plane lattice parameter (Å)	3.87	3.87	3.83
Mean surface roughness (nm)	0.36	0.21	0.52

**Figure A1:**
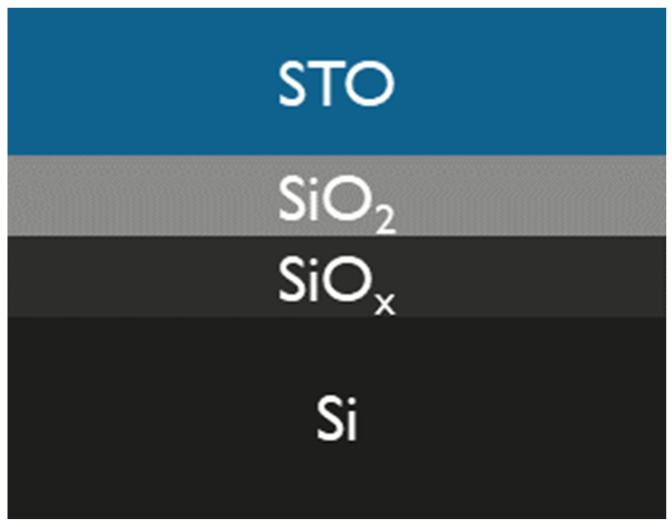
Layer stack used in ellipsometry fitting model.

**Figure A2:**
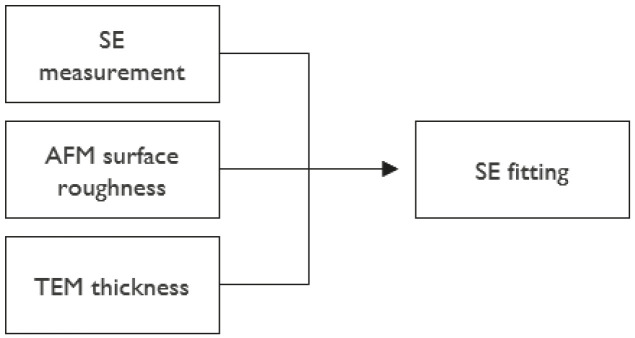
Fitting procedure for ellipsometry results.

## Data Availability

Data are contained within the article.

## Appendix A

Five Tauc–Lorentz oscillators were used to build the ellipsometer fitting model. Figure A1 schematically shows the considered layer stack, which includes both an intermediate silicon oxide layer for modeling the silicon oxidation SiO_x_ and a SiO_2_ layer between the STO and Si.

To ensure accurate fitting results, the surface roughness and the thicknesses of the STO and SiO_2_ layers were determined using AFM and TEM, respectively, and then incorporated into the analysis. Finally, the fit was optimized by adjusting the fitting boundaries as shown in Figure A2.

The mean square error (MSE) is calculated with following formula by the CompleteEASE software (version 6.65):

$$MSE=\sqrt{\frac{1}{3n-m}\sum_{i=1}^{n}\left[{\left(\frac{N_{E_{i}}-N_{G_{i}}}{0.001}\right)}^{2}+{\left(\frac{C_{E_{i}}-C_{G_{i}}}{0.001}\right)}^{2}+{\left(\frac{S_{E_{i}}-S_{G_{i}}}{0.001}\right)}^{2}\right]}$$

where *n* is the number of wavelengths; *m* is the number of fitting parameters; and *N* = cos(2*Ψ*), *C* = sin(2*Ψ*) cos(Δ), *S* = sin(2*Ψ*) sin(Δ). Subscript *E* and *G* denote measured and generated data, respectively. For all our samples, MSE values are below 6, indicating a very good fit as illustrated in Table A1. The annealed Sr-rich STO sample was fitted with slightly larger error, which could be due to formation of an additional Sr-rich SiO_x_ layer at the interface, as seen in Figure 5c.

**Table A1:** Overview of parameters used in ellipsometry fitting and MSE values.

	Thickness of the Layers (nm):			Mean Surface Roughness (nm):	MSE
	STO	SiO_2_	SiO_x_		
Ti-rich STO					
as-grown:	16.8	0.0	0.2	0.36	2.11
30 min annealed:	17.6	3.2	0.9	0.46	2.43
Stoichiometric STO					
as-grown:	13.0	1.7	1.2	0.21	2.92
30 min annealed:	14.0	3.9	1.5	0.19	3.10
Sr-rich STO					
as-grown:	18.8	0.0	0.5	0.52	3.25
30 min annealed:	18.6	2.8	1.5	0.23	5.67
